# Overexpression of Catalase Enhances Benzo(a)pyrene Detoxification in Endothelial Microsomes

**DOI:** 10.1371/journal.pone.0162561

**Published:** 2016-09-08

**Authors:** Fang Yang, Hong Yang, Aramandla Ramesh, J. Shawn Goodwin, Emmanuel U. Okoro, ZhongMao Guo

**Affiliations:** 1 Department of Physiology, Meharry Medical College, Nashville, United States of America; 2 Wuhan University School of Basic Medical Science, Wuhan, P.R. China; 3 Department of Biochemistry and Cancer Biology, Meharry Medical College, Nashville, United States of America; Universite Paris Diderot, FRANCE

## Abstract

We previously reported that overexpression of catalase upregulated xenobiotic- metabolizing enzyme (XME) expression and diminished benzo(a)pyrene (BaP) intermediate accumulation in mouse aortic endothelial cells (MAECs). Endoplasmic reticulum (ER) is the most active organelle involved in BaP metabolism. To examine the involvement of ER in catalase-induced BaP detoxification, we compared the level and distribution of XMEs, and the profile of BaP intermediates in the microsomes of wild-type and catalase transgenic endothelial cells. Our data showed that endothelial microsomes were enriched in cytochrome P450 (CYP) 1A1, CYP1B1 and epoxide hydrolase 1 (EH1), and contained considerable levels of NAD(P)H: quinone oxidoreductase-1 (NQO1) and glutathione S-transferase-pi (GSTP). Treatment of wild-type MAECs with 1μM BaP for 2 h increased the expression of microsomal CYP1A1, 1B1 and NQO1 by ~300, 64 and 116%, respectively. However, the same treatment did not significantly alter the expression of EH1 and GSTP. Overexpression of catalase did not significantly increase EH1, but upregulated BaP-induced expression of microsomal CYP1A1, 1B1, NQO1 and GSTP in the following order: 1A1>NQO1>GSTP>1B1. Overexpression of catalase did not alter the distribution of each of these enzymes in the microsomes. In contrast to our previous report showing lower level of BaP phenols versus BaP diols/diones in the whole-cell, this report demonstrated that the sum of microsomal BaP phenolic metabolites were ~60% greater than that of the BaP diols/diones after exposure of microsomes to BaP. Overexpression of catalase reduced the concentrations of microsomal BaP phenols and diols/diones by ~45 and 95%, respectively. This process enhanced the ratio of BaP phenol versus diol/dione metabolites in a potent manner. Taken together, upregulation of phase II XMEs and CYP1 proteins, but not EH1 in the ER might be the mechanism by which overexpression of catalase reduces the levels of all the BaP metabolites, and enhances the ratio of BaP phenolic metabolites versus diol/diones in endothelial microsomes.

## Introduction

Benzo(a)pyrene (BaP), a polycyclic aromatic hydrocarbon (PAH) compound, has been shown to contribute to the development of atherosclerosis-related cardiovascular disease [1, 2]. The atherogenic role of BaP is due to its reactive intermediates [3–5] and reactive oxygen species (ROS) generated during its metabolism [6–8]. The level of BaP reactive intermediates and ROS is controlled by the coordinated activity of phase I and phase II xenobiotic-metabolizing enzymes (XMEs). Specifically, phase I enzymes, such as cytochrome P450 (CYP)-1 family proteins and epoxide hydrolase 1 (EH1), catalyze the formation of BaP reactive intermediates, while phase II enzymes, such as glutathione S-transferases (GSTs), UDP glucuronosyl-transferases (UGTs) and sulfotransferases (SULTs), detoxify BaP intermediates by converting them to less reactive and water soluble conjugates [9, 10], which are exported out of the cells and finally excreted through the urine and feces. In addition, phase II enzymes NAD(P)H: quinone oxidoreductase-1 (NQO1) prevents the redox cycling of BaP quinone-semiquinone-quinols, thus reducing ROS generation.

Among the three members of CYP1 enzymes, CYP1A1 and 1B1 are best known for PAH metabolism [11]. It has been shown that elimination of hepatic CYP function by knockout of CYP reductase increased BaP-DNA adducts in mouse liver [12]. The formation of these adducts imply a more important role of hepatic CYP1 proteins in BaP detoxification than in its bioactivation. Increasing evidence suggests that the detoxification activity of CYP1 proteins results primarily from the 1A1 isoenzyme. Specifically, knockout of CYP1A1 augments BaP-DNA adducts and BaP-induced toxicity [13], while knockout of CYP1B1 results in protection against PAH-induced toxicity in mice [14]. The mechanism underlying these contradictory results has not been fully elucidated. One possibility is that the metabolites generated by CYP1A1 and 1B1 are different, *i*.*e*., CYP1A1 is more efficient in the conversion of BaP to hydroxyl, quinone, and diol metabolites, whereas CYP1B1 is more competent in the formation of more complicated intermediates, such as BaP-7,8-dihydrodiol- 9,10-epoxide [15], which binds covalently to proteins, lipids and DNA. Thus, a balance between expression of CYP1A1 and 1B1 enzymes governs BaP-induced toxicity.

Another explanation for the different effects of CYP1 proteins on BaP metabolism is that CYP1A1 is tightly coupled to phase II enzymes in the endoplasmic reticulum (ER). The close spatial association or geographically tight coupling of phase I and phase II XMEs on the ER membrane would facilitate the sequential reactions of BaP bioactivation and detoxification, preventing accumulation of reactive intermediates in the cells. In contrast, CYP1B1 may not be tightly coupled to phase II XMEs, resulting in the BaP intermediates accumulation [16]. While this theory is promising, it has not been tested yet.

We previously reported that overexpression of antioxidant enzyme catalase diminished BaP-induced atherosclerosis in hypercholesterolemic mice [4], reduced BaP intermediates [17], and enhanced BaP-induced expression of NQO1, GST-pi (GSTP), CYP1A1 and 1B1 in mouse aortic endothelial cells (MAECs) [17, 18]. We also observed that the level of CYP1A1 increased by BaP exposure and catalase overexpression was greater than that of CYP1B1. The upregulatory activity of catalase on NQO1, GSTP, and CYP1A1 was mediated by a mechanism involving aryl hydrocarbon receptor (AhR), and the catalase-increased CYP1B1 expression was mediated by an AhR-independent pathway, as knockdown of AhR diminished BaP-increased NQO1, GSTP, and CYP1A1 expression, but did not significantly alter BaP-induced CYP1B1 in cells overexpressing catalase [17].

The present report described the effect of overexpressing catalase in MAECs on the levels of microsomal XMEs and BaP intermediates. Our data demonstrated that CYP1A1, CYP1B1 and EH1 were located primarily in the microsomes. Though historically thought as cytosolic proteins, considerable levels of NQO1 and GSTP were also found in the microsomes. Exposure of MAECs to BaP upregulated the expression of microsomal CYP1A1, CYP1B1, NQO1 and GSTP, but not EH1. Overexpression of catalase reduced BaP intermediates, and upregulated BaP-induced expression of these XMEs in an order as follows: 1A1>NQO1>GSTP>1B1>EH1. Overexpression of catalase did not alter the distribution of these XMEs in microsomes. Thus, decrease in microsomal BaP intermediates in catalase-overexpressing cells unlikely result from an increased spatial association of phase I and phase II XMEs, but might result from an increased expression of XMEs in a way that shifts the balance between CYP1A1 and 1B1 and the balance between phase I and phase II XMEs in favor of reducing BaP intermediates.

## Materials and Methods

### Isolating and culturing mouse aortic endothelial cells (MAECs)

Transgenic mice overexpressing human catalase were generated by injection of fertilized 57BL/6 embryos with a fragment of human genomic DNA containing the entire human catalase gene as described previously [19]. The catalase activity in the aorta [19], endothelial cells [20] and smooth muscle cells (SMCs) [21] obtained from mice hemizygous for human catalase transgene (*hCat*Tg) was approximately 2.5-fold higher than in wild-type (WT) littermates. In addition, the catalase activity in other tissues, including heart, kidney, lung, liver, muscle and spleen, obtained from the *hCat*Tg mice was 2- to 4-fold higher than in those obtained from WT controls. In contrast, the activities of other antioxidant enzymes, such as Cu/Zn-superoxide dismutase (SOD), Mn-SOD, extracellular-SOD, and glutathione peroxidase-1 were comparable in tissues and cells obtained from WT and *hCat*Tg mice [19]. Mouse aortic endothelial cells (MAECs) were obtained from *hCat*Tg and wild-type mice at 3 to 4 months of age using an outgrowth technique [20]. Freshly isolated MAECs displayed a cobblestone-like monolayer and expressed von Willebrand factor and platelet-endothelial cell molecule-1 (CD31) [20]. These characteristics remain unchanged over 15 passages (data not shown). The 8^th^ and 9^th^ passages of MAECs were used in this report. All procedures for handling animals were conducted following protocols approved by the Institutional Animal Care and Use Committee at Meharry Medical College.

### Measurement of reactive oxygen species (ROS)

For detection of intracellular ROS, MAECs were grown to confluence in a black-walled bottom-clear 96-well plate in Dulbeco’s Modified Eagle’s Medium (DMEM) supplemented with 10% fetal bovine serum (FBS) and 1% Penicillin/Streptomycin at 37°C in a 95% air and 5% CO_2_ atmosphere, and then incubated for 1 h with 10 μg/ml 6-carboxy-2',7'-dichlorodi- hydrofluorescein diacetate (CDC-H2F diacetate) (Molecular Probes Inc., Eugene, OR). After three washes with Hank’s buffer, 100 μl of serum-free DMEM with or without 1 μM BaP (Sigma-Aldrich, St. Louis, MO) was added to each well and incubated for 30 min. Fluorescence was read using a fluorometer (Fluoroskan Ascent FL, ThermoLabsystems, Philadelphia, PA) with excitation/emission wavelengths of 480/540 nm for CDC-H2F.

For measurement of hydrogen peroxide (H_2_O_2_) release, MAECs grown to confluence in a 96-well plate were incubated with 150 μl Amplex red reagent (Invitrogen, Carlsbad, CA) in the presence or absence of 1 μM BaP for 30 min at 37°C. Fluorescence was read using a Fluoskan Ascent fluorometer with the excitation and emission wavelengths at 540 and 590 nm respectively. The cumulative H_2_O_2_ concentration was determined based on the standard curve obtained by incubation of the Amplex red reagent with H_2_O_2_. Because this measurement reflects the ability of MAECs to release H_2_O_2_, we refer to it as H_2_O_2_ release. At the end of the experiments, MAECs were lysed in M-PER mammalian protein extraction reagent (Thermo Scientific, Rockford, IL). Protein levels in the lysate were determined using a BCA protein assay kit (Thermo Scientific). The levels of H_2_O_2_ were expressed relative to the protein levels.

### Western blot analysis

MAECs grown in 100-mm culture dishes were treated with 100 μl of serum-free DMEM containing 1 μM BaP or 2 μl DMSO for 4 h. For whole-cell protein extraction, cells were lysed in M-PER mammalian protein extraction reagent. Microsomes were prepared as described previously [22, 23]. Briefly, MAECs were harvested by trypsin/EDTA digestion, and homogenized with a Dounce homogenizer in a lysis buffer [20% glycerol, 10mmol/L Tris-HCl (pH 7.4), 1 mM EDTA (pH 7.4), 1 mM DTT]. The homogenate was centrifuged serially at 1,000 g for 10 min and at 12,000 g for 15 min to remove cell debris, nuclei and mitochondria in the pellets. The resulting supernatant was centrifuged at 100,000 g for 60 min in a Beckman Optima L-80 XP ultracentrifuge using a Ti70 rotor (Beckman Coulter, Brea, CA). All the procedures were performed at 4°C. The pellet containing microsomes was collected in M-PER mammalian protein extraction reagent. The whole-cell and microsomal lysates were resolved on a 10% sodium dodecyl sulfate (SDS)-polyacrylamide gel. Proteins were transferred to a PVDF membrane. After blocking with 3% fat-free milk, the membranes were incubated sequentially with primary and secondary antibodies. The antibodies against β-actin (sc130656), protein disulfide isomerase (PDI) (sc20132), catalase (sc50508), CYP1A1 (sc48432), CYP1B1 (sc32882), EH1 (sc22748), NQO1 (sc16464) and GSTP (sc134469) were obtained from Santa Cruz Biotechnology (Santa Cruz, CA). Immunoreactive bands were visualized using ECL-plus chemiluminescence reagent (GE Healthcare-Amersham, Piscataway, NJ) and analyzed with a GS-700 Imaging Densitometer (Bio-Rad, Hercules, CA) [17]. The uneven sample loading was normalized using the intensity ratio of the immunoreactive bands of the tested proteins relative to β-actin.

### High-performance liquid chromatography (HPLC) analysis for BaP intermediates

MAECs were treated for 4 h with 10 nM of 2,3,7,8-tetrachlorodibenzo-p-dioxin (TCDD) (Sigma-Aldrich), an AhR ligand that has been shown to induce XME expression [24]. Microsomes were isolated from TCDD-pretreated MAECs by serial centrifugations as described above, and suspended in 120 μl serum-free DMEM (final protein concentration 0.5 mg/ml) containing 1 μM BaP. The reaction was initiated by adding 5 ml of cocktail containing NADPH (0.72 mM), EDTA (100 mM), KPO_4_ (100 mM), and MgCl_2_ 6H_2_O (3.75 mM). After incubation at 37°C for 1 h, the reaction was stopped with 8 ml ethyl acetate containing butylated hydroxytoluene (0.2 mg/ml) [22]. The microsomal suspension was mixed with 0.1% SDS, and extracted with a solution containing water, methanol and chloroform at a ratio of 1:1.5: 2 (v/v). The organic phase was dried under N_2_ and resuspended in 0.5 ml of methanol. Particulates in the extracts were removed by passing them through Acrodisc filters (0.45 μm, 25 mm diameter; Gelman Sciences, Ann Arbor). The final extracts were stored at 4°C in amber color screw top vials to prevent photo-degradation until analyzed. The extracts were analyzed using a HPLC system (Agilent Technologies, Wilmington, DE) equipped with a C_18_ reverse-phase column (Octadecylsilane Hypersil, 5 μm, 200 × 4.6 mm; Agilent Technologies) and hooked to a UV detector (scanned at 254 nm; Agilent Technologies). Elution was performed with a linear gradient from 40% methanol to 100% methanol in 45 min at a flow rate of 1.0 ml/min [25]. Benzo(a)pyrene metabolite standards were purchased from the National Cancer Institute Chemical Carcinogen Repository (Midwest Research Institute, Kansas City, MO). Identification of the metabolites was accomplished by comparison of retention times and peak areas of the samples with that of standards.

### Fluorescence Microscopic Analysis of CYP1A1 and 1B1

MAECs grown on chamber slides were treated with 1 μM BaP or medium alone at 37°C for 4 h. Following three washes with phosphate buffered saline (PBS), the cells were fixed with 3.7% paraformaldehyde at room temperature for 15 min, and permeabilized with 0.1% Triton-X 100 at room temperature for 20 min. After blocking with 5% goat serum, the slides were incubated with antibodies against CYP1A1 or CYP1B1, and then incubated with Texas Red goat anti-mouse IgG_1_ secondary antibody for CYP1A1 or Alexa Fluor 568 donkey anti-rabbit secondary antibody for CYP1B1. Thereafter, the cells were incubated for 10 min with 250 nM of Tio6(3) (Invitrogen, Carlsbad, CA), which permeates the cellular membrane and is commonly used as a probe to localize endoplasmic reticulum. Following three rinses, the slide was mounted with a glass coverslip with ProLong mounting medium (Invitrogen), and viewed using a Nikon A1R confocal microscope (Nikon Instruments, Melville, NY). The two channels were acquired sequentially with the following excitation and emission parameters: Tio6(3) was excited at 488 nm with an argon laser and fluorescence was detected from 500–550 nm. Texas Red and Alexa Fluor 568 were excited with the 561 nm diode laser and fluorescence was detected at 570–620 nm. High-resolution (100 nm/pixel) Z-series images were obtained from MAECs with a ×60 (1.4 N.A.) plan apochromat oil-immersion objective and analyzed for co-localization. Overlay images were assembled, zoomed, and cropped using Nikon Elements 2.3 software.

### Statistical analysis

Data are reported as the mean ± SEM. Differences among control and treatment groups were analyzed by Student’s unpaired *t*-test (for two groups) and one-way or multiple factor analysis of variance (for more than two groups) followed by Tukey’s post-hoc test. Statistical significance was considered when *P* was less than 0.05. For the experiments using the 96 well microplate reader, the mean value for each experiment was averaged from triplicate wells in the same plate. The number of experiments was indicated in figure legends. VassarStats (vassarstats.net) software was used for statistical analysis.

## Result

### Overexpression of catalase reduces peroxide radicals in MAECs

**Fig 1:**
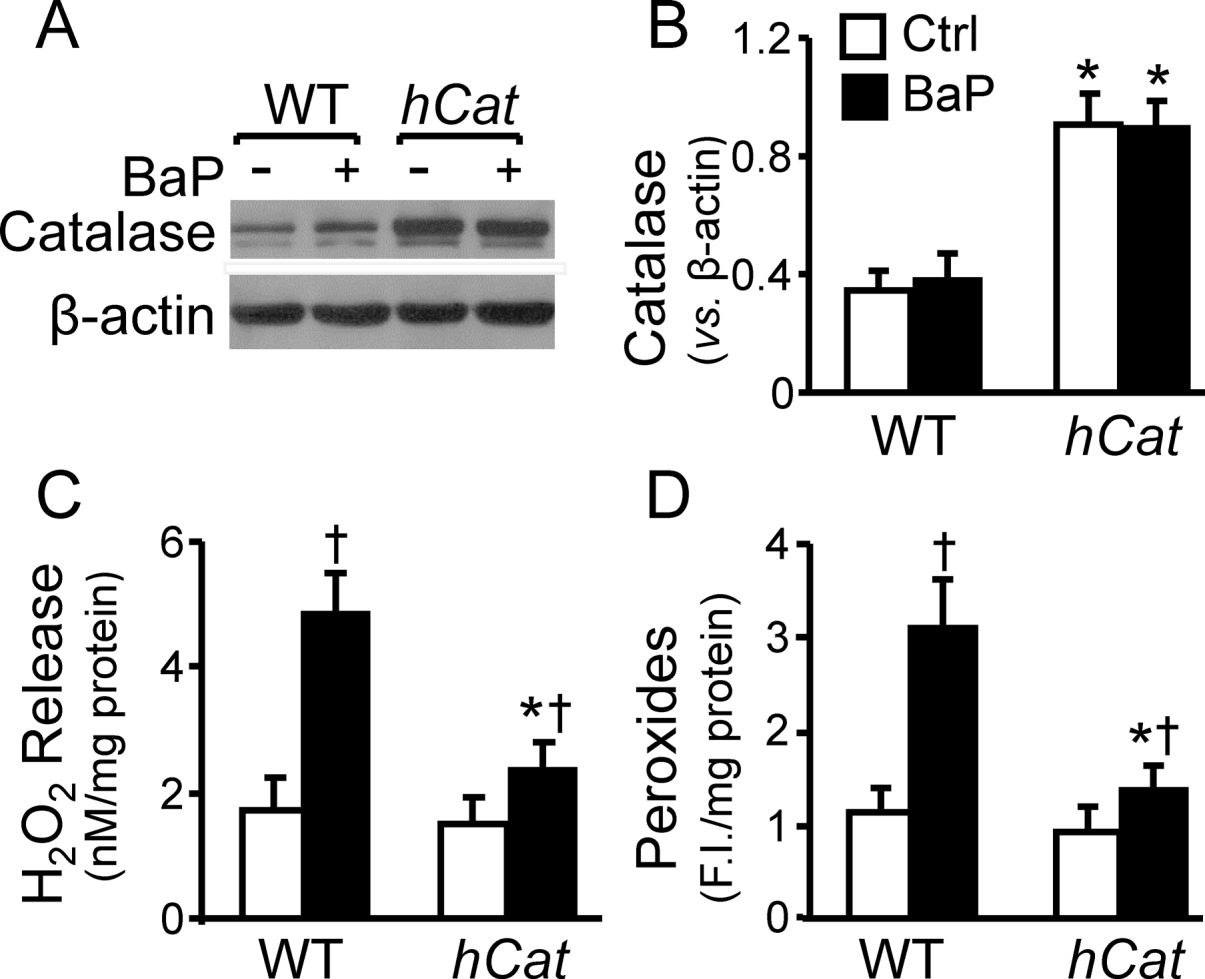
Overexpression of catalase reduces BaP-induced peroxides. MAECs obtained from wild-type (WT) mice and transgenic mice overexpressing human catalase (*hCat*) were incubated with 1 μM of BaP for 30 min or culture medium alone as a control (Ctrl). The catalase protein level was determined in whole-cell extracts by western blot analysis (A) and quantified relative to β-actin (B). The release of H_2_O_2_ from MAECs (C) were measured using an Amplex red hydrogen peroxide assay kit, and the intracellular level of peroxide radicals (D) was measured using CDC-H2F diacetate as a probe. Values represent the mean ± SEM of three separate experiments; MAECs were pooled from 4 mice. * *P<0*.*05 vs*. WT cells with same treatments, and ^†^ *P<0*.*05 vs*. the same genotype cells without BaP treatment.

We previously reported that that endothelial cells obtained from *hCat*Tg mice had ~2.5 fold increase in their catalase activity, and no significant change in the activities of other antioxidant scavengers, including Cu/Zn-superoxide dismutase (SOD), Mn-SOD, extracellular-SOD, and glutathione peroxidase-1, when compared with the cells obtained from wild-type (WT) littermates [20]. Data from the present study show that the catalase protein level were about 2.6 fold higher in *hCat*Tg MAECs than in WT cells, and that BaP barely altered the catalase protein level in both *hCat*Tg and WT MAECs (Fig 1A and 1B). Data in Fig 1C and 1D also show that under control conditions the release of H_2_O_2_ and the level of intracellular peroxides were slightly lower in *hCat*Tg MAECs than in WT cells, but the differences were not statistically significant. In contrast, *hCat*Tg and WT MAECs show significantly different responses to BaP with regard to H_2_O_2_ release and intracellular peroxide level. Thus, addition of 1 μM BaP to the culture medium elevated H_2_O_2_ release and cellular peroxide level by ~2.8 and 2.7 fold in WT MAECs, respectively. However, the same dose of BaP induced only ~1.6 and 1.5 increases in H_2_O_2_ release and cellular peroxide level in *hCat*Tg cells.

### Overexpression of catalase enhances BaP-induced expression of XMEs in endothelial microsomes

**Fig 2:**
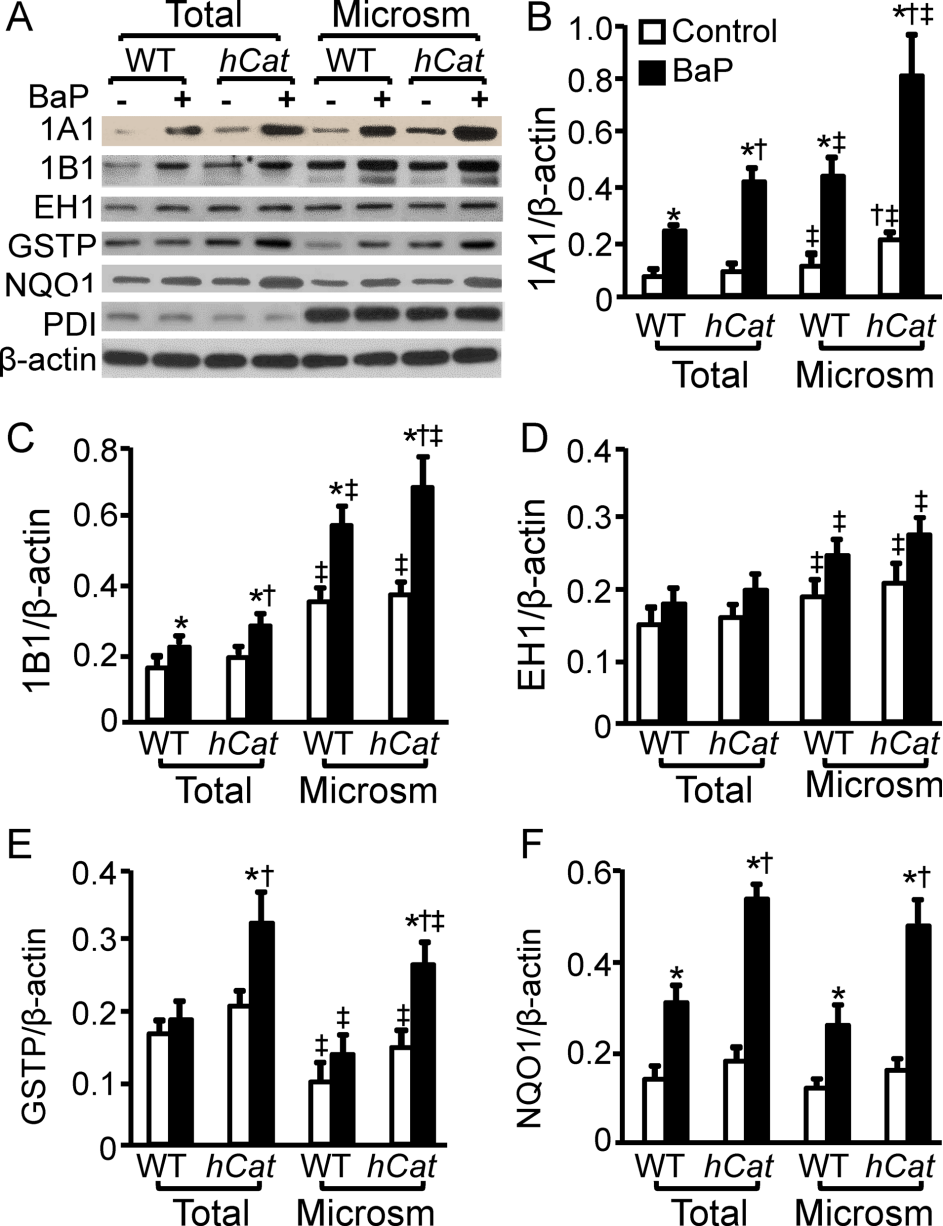
Overexpression of catalase enhances expression of BaP-induced xenobiotic- metabolizing enzymes in endothelial microsomes. MAECs obtained from wild-type (WT) mice and transgenic mice overexpressing human catalase (*hCat*) were incubated with 1 μM of BaP or culture medium alone as a control for 4 h. Microsomes were isolated from MAECs with serial centrifugations. The total and microsomal levels of CYP1A1, EH, GSTP, NQO1 and PDI were determined by western blot analysis, and expressed as a ratio of their immunoblot intensity relative to β-actin. Values represent the mean ± SEM of five separate experiments in which MAECs were pooled from 4 mice. * *P*< 0.05 *vs*. control; ^†^ *P*< 0.05 *vs*. BaP-treated WT cells; and ^‡^ *P*< 0.05 *vs*. the total level of the protein in the same genotype cells with BaP treatment.

The crude microsomal preparation obtained by the serial centrifugations contain both rough and smooth ER [26]. The images in Fig 2A show that the β-actin level was comparable in the whole-cell lysates and the microsomal preparations; while the level of PDI, a protein enriched in ER, was ~6 fold higher in the microsomal preparations than in the whole-cell lysates. These suggest that the crude microsomal preparations obtained from MAECs are enriched in ER proteins. Treatment with BaP and/or overexpression of catalase did not significantly affect the protein levels of β-actin and PDI in MAECs.

We previously reported that overexpression of catalase upregulated BaP-induced expression of phase I enzymes CYP1A1 and CYP1B1, and phase II enzymes NQO1 and GSTP by activation of AhR. The present study determined the effect of catalase overexpression on the distribution and expression level of these proteins and another phase I enzyme EH1 in microsomes. As the data in Fig 2 show, the basal levels of CYP1A1, 1B1 and EH1 were ~1.6, 2.3 and 1.3 fold higher, respectively, in the microsomal preparations than in the whole-cell lysates. The NQO1 level in the whole-cell lysates and microsomes was comparable, while the GSTP level was ~30% lower in the microsomes under the control conditions. The data in Fig 2B also show that the basal level of CYP1A1 was slightly higher in the whole-cell lysates but significantly higher in the microsomes obtained from *hCat*Tg MAECs than in those obtained from WT cells. Overexpression of catalase did not significantly alter the basal levels of CYP1B1, EH1, GSTP and NQO1, and thus no significant difference was observed between WT and *hCat*Tg MAECs under the control conditions (Fig 2C–2F).

Exposure of MAECs to BaP (1 μM) slightly increased the EH1 level; however, the difference between cells treated with and without BaP did not reach statistical significance (Fig 2D). In contrast, BaP treatment increased the total and microsomal levels of other proteins studied. The magnitude of increase varied from protein to protein. Specifically, 1 μM BaP exposure for 2 h elevated the total CYP1A1 protein level by 3.4 and 4.7 fold, and its microsomal level by ~4 and 4 fold in WT and *hCat*Tg MAECs, respectively (Fig 2B). As a consequence of the upregulatory effect of catalase overexpression on CYP1A1 expression, the total and microsomal CYP1A1 proteins were ~2 fold higher in *hCat*Tg than in WT cells after BaP treatment (Fig 2B). As the data in Fig 2C show, the expression of CYP1B1 induced by BaP treatment and catalase overexpression was relatively lower compared to CYP1A1. Specifically, BaP treatment increased the total CYP1B1 by ~40 and 50% and the microsomal CYP1B1 by 64 and 86%, respectively, in WT and *hCat*Tg MAECs. The total and microsomal CYP1B1 proteins in *hCat*Tg MAECs were significantly higher than those in WT cells after treatment with BaP (Fig 2C).

The data in Fig 2E show that treatment with 1 μM BaP for 2 h increased total and microsomal GSTP proteins in *hCat*Tg cells by ~60 and 86%, respectively; but did not significantly alter the GSTP protein level in wild-type MAECs. The same dose of BaP induced NQO1 in both WT and *hCat*Tg MAECs, and the induced magnitude was significantly greater in *hCat*Tg than in WT cells. Specifically, BaP enhanced both total and microsomal NQO1 proteins ~3 fold in *hCat*Tg MAECs, but only about 2 fold in WT cells, leading to a significantly higher level of NQO1 proteins in the *hCat*Tg MAECs than in WT cells following BaP treatment (Fig 2F).

### Fluorescence microscopy for subcellular localization of CYP1A1 and 1B1

**Fig 3:**
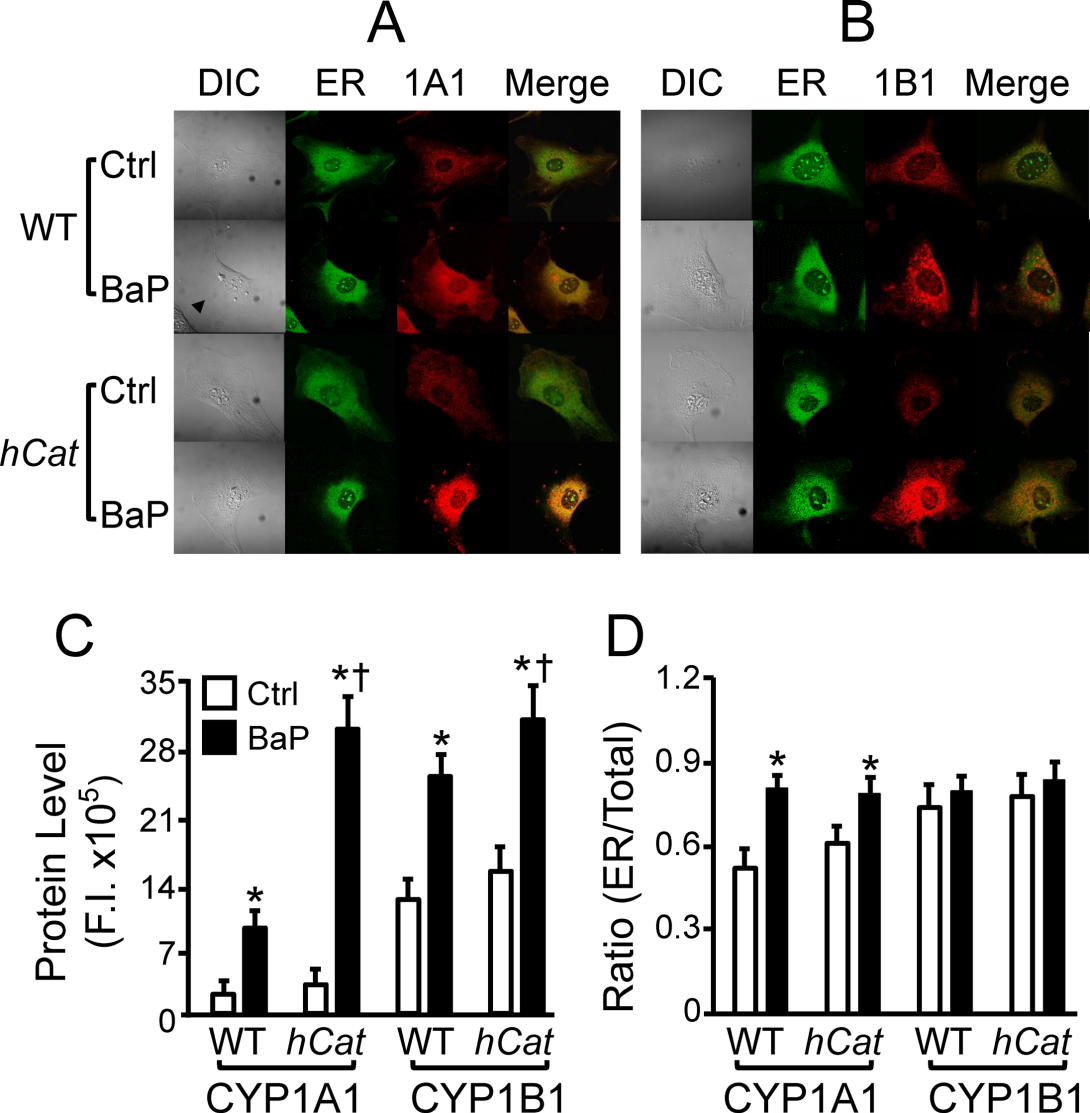
Localization of CYP1A1 and 1B1 in endoplasmic reticulum. MAECs obtained from wild-type (WT) mice and transgenic mice overexpressing human catalase (*hCat*) were incubated with 1 μM of BaP or culture medium alone as a control (ctrl) for 4 h. After the cells were fixed and permeabilized, they were immunostained with antibodies against CYP1A1 (1A1) or CYP1B1 (1B1), and then stained with endoplasmic reticulum (ER) marker Tio6(3). (A-B) Images were obtained using a Leica TCS SP2 confocal microscope. (C) The protein levels of CYP1A1 and 1B1 were determined by the fluorescence intensity (F.I.). (D) The ratio of CYP1A1 and 1B1 proteins in the ER versus those in the whole-cell was determined by the F.I. in the ER and the entire cell. * *P*< 0.05 *vs*. the same genotype cells without BaP treatment (ctrl); and ^†^ *P*< 0.05 *vs*. BaP-treated WT cells.

This study confirmed the ER distribution of CYP1A1 and 1B1 using a fluorescence microscopy. These CYP1 proteins were stained with antibodies conjugated with red fluorescence, and the ER was stained with a green fluorescence dye Tio6(3). The colocalization of CYP1 proteins in the ER was determined by the yellow fluorescence in merged images. The data in Fig 3 show that the basal level of CYP1A1 is lower as compared to CYP1B1. Treatment with BaP and overexpression of catalase increased the expression of these proteins, as reflected by the increased fluorescence intensity. Consistent with the western blot data shown in Fig 2, the BaP- and catalase-induced changes vary between CYP1A1 and 1B1. Thus, treatment of the WT MAECs with 1 μM BaP for 2 h induced ~4.5 and 2 fold increase in the protein levels of CYP1A1 and 1B1, respectively. The same BaP exposure concentration (dose) and duration of BaP treatment elevated the expression of these proteins by ~10 and 2 fold in *hCat*Tg MAECs (Fig 3B). Thus, the BaP-induced expression of CYP1A1 is significantly higher in *hCat*Tg than in WT MAECs.

The images in Fig 3 show that both CYP1A1 and 1B1 are distributed in the ER and extra-ER compartments; however, the ratio of CYP1A1 in the ER and the whole-cell is lower than that of CYP1B1 under the control condition. Treatment with BaP significantly elevated the ratio of ER and total CYP1A1, but did not alter the ratio of CYP1B1. Overexpression of catalase did not significantly affect the distribution of CYP1A1 and 1B1 in the ER either before or after BaP treatment. Specifically, treatment with 1 μM BaP for 2 h elevated the ratio of ER and total CYP1A1 from 0.52 to 0.82 in WT cells, and from 0.62 to 0.8 in *hCat*Tg cells, respectively (Fig 3D). The ratio of ER and total Cyp1B1 is 0.75–0.82 in WT and *hCat*Tg MAECs treated with or without BaP (Fig 3D). No significant difference was observed in WT and *hCat*Tg MAECs in the absence or presence of BaP treatments.

### Overexpression of catalase reduces BaP intermediates in endothelial microsomes

**Fig 4:**
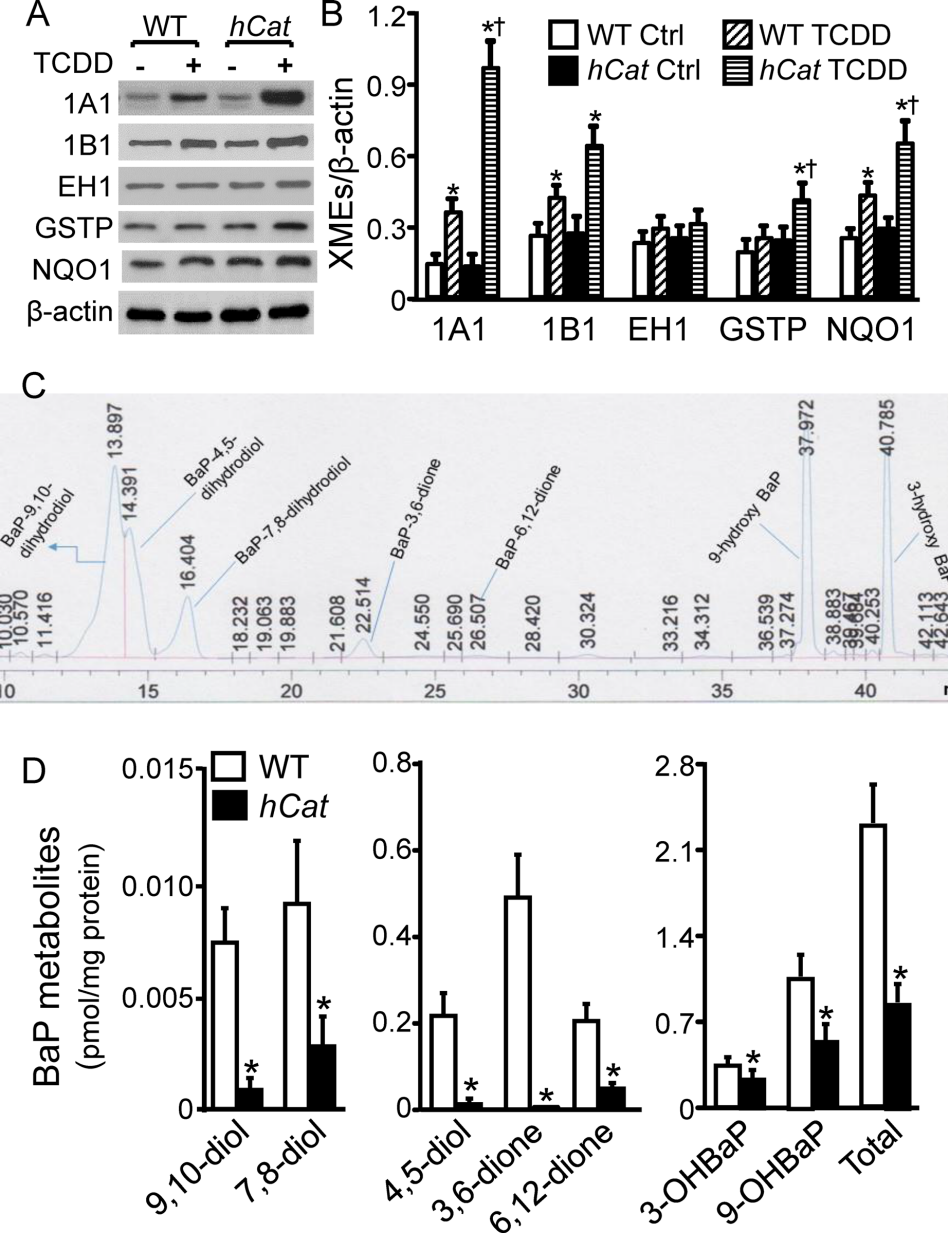
Overexpression of catalase reduces BaP reactive intermediates in endothelial microsomes. (A-B) MAECs obtained from wild-type (WT) mice and transgenic mice overexpressing human catalase (*hCat*) were incubated with 10 nM of TCDD for 4 h. Microsomes were isolated from TCDD-treated MAECs with serial centrifugations. The total and microsomal levels of CYP1A1, EH, GSTP and NQO1 were determined by western blot analysis, and expressed as a ratio of their immunoblot intensity relative to β-actin. (C-D) Microsomes were isolated from TCDD-treated MAECs with serial centrifugations, and incubated with 1 μM of BaP for 1 h. BaP reactive metabolites, including 9-hydroxybenzo[a]pyrene (9-OH BaP), 3-hydroxy BaP(3-OH BaP), 9,10-dihydroxy BaP (9,10-diol), 7,8-dihydro BaP (7,8-diol), 4,5-dihydroxy BaP (4,5-diol), BaP-3,6-dione (3,6-dione) and BaP-6,12-dione (6,12-dione), in the microsomes were determined using a reverse phase HPLC system. Panel C shows a representative HPLC chromatogram of BaP metabolites, and panel D shows the level of BaP metabolites as expressed relative to the level of microsomal proteins. Values represent the mean ± SEM of five separate experiments in which MAECs were pooled from 4 mice. * *P*< 0.05 *vs*. the same genotype cells without TCDD treatment (control), and ^†^ *P*< 0.05 *vs*. WT cells treated with TCDD.

Since overexpression of catalase did not significantly alter the basal level of XMEs in microsomes (Fig 2), here we stimulated XME expression by pretreatment of MAECs with 10 nM TCDD, and studied the effect of catalase overexpression on BaP metabolism in the microsomes obtained from TCDD-treated cells. The data in Fig 4A show that TCDD exposure elevated CYP1A1, 1B1, GSTP and NQO1 protein levels by 7.5, 2.3, 1.7 and 2.4 fold, respectively, in the microsomes obtained from *hCat*Tg MAECs, and 2.6, 1.6, 1.3 and 1.7 fold, respectively, in the microsomes obtained from WT cells. Similar to the observations for BaP-induced XME expression, TCDD-induced expression was greater in the transgenic than in WT cells. A slight increase in microsomal EH1 level was observed in TCDD-treated MAECs compared to untreated controls; however, the difference between TCDD-treated and untreated cells was not statistically significant (Fig 4A and 4B).

The data in Fig 4B show that treatment of microsomes with 1 μM BaP for 4 h induced detectable accumulation of BaP reactive intermediates, including 9-hydroxybenzo[a]pyrene (9-OH BaP), 3-hydroxy BaP (3-OH BaP), 9,10-dihydroxy BaP (9,10-diol), 7,8-dihydro BaP (7,8-diol), 4,5-dihydroxy BaP (4,5-diol), BaP-3,6-dione (3,6-dione) and BaP-6,12-dione (6,12-dione). The sum of the two BaP phenols 9-OH BaP and 3-OH BaP was ~1.6 fold higher than the sum of the BaP diols and diones in WT MAECs. The sum of phenols, the sum of diols/diones, and the total level of BaP intermediates studied were reduced by ~45, 95 and 63%, respectively, in the microsomes obtained from *hCat*Tg MAECs compared to that obtained from WT cells (Fig 4). The more profound reduction in BaP diols/diones than in phenols resulted in the difference of these two classes of BaP intermediates even larger. Thus, the sum of BaP phenolic metabolites was ~13 fold greater than diols/diones in *hCat*Tg cells.

## Discussion

Endoplasmic reticulum (ER), including the rough and smooth ER, is the most active organelle involved in BaP metabolism, though other subcellular compartments such as the mitochondria and cytosol, are also able to metabolize BaP [27]. The present report demonstrated that the sum of 9(OH)- and 3(OH)BaP generated by the microsomes was ~1.6 fold higher than that of the BaP diols/diones in WT MAECs. In contrast, we previously observed that these phenolic metabolites in the whole-cell were ~2.6 fold lower than the diols/diones in WT MAECs [17]. These findings suggest that BaP might be metabolized in the ER and other subcellular compartments as well. The metabolites generated from the ER are mainly BaP phenols, while those from the extra-ER compartments are mainly BaP diols/diones. It is known that BaP intermediates are generated by CYP1 proteins and EH1. Specifically, CYP1 proteins oxidize BaP to form BaP phenols and epoxides. The phenolic metabolites are further oxidized to quinones by CYP1 proteins, while the epoxides are hydrolyzed by EH1 to form BaP diols, which can be autooxidized to diones or converted by CYP1 proteins to tetrols, such as BaP-7,8-dihydrodiol-9,10-epoxide [28]. It has been reported that in the absence of EH1, BaP was metabolized primarily to phenols and quinones in microsomes. Addition of a purified EH1 into the microsomal suspension resulted in the formation of BaP diols with a concomitant decrease in the formation of phenolic metabolites [29]. These observations suggest that the relative level of EH1 versus CYP1 proteins controls the contribution of the phenol/quinone and the epoxide/diol pathways in BaP bioactivation. The three members of CYP1 proteins, *i*.*e*., CYP1A1, 1A2 and 1B1, are located primarily in the ER [30], with modest distribution in other subcellular compartments, such as mitochondria, plasma membrane and lysosomes [31, 32]. Two EH isoenzymes have been reported in mammalian cells. EH1 is located primarily in the rough ER and has been shown to hydrolyze BaP epoxides [33]; while EH2 is enriched in several extra-ER compartments, such as the mitochondria, peroxisomes and cytosol, and has not been reported to hydrolyze PAH metabolites, instead playing a major role in metabolism of endogenous lipid epoxides [34, 35]. Data from this report suggest that CYP1A1, 1B1 and EH1 were located largely in the microsomes of MAECs. Further studies are needed to determine the ratio of EH1 versus CYP1 proteins in the ER and other subcellular compartments. It is possible that though CYP1A1, 1B1 and EH1 are located mainly in the ER, the ratio of EH1 versus CYP1 proteins is lower in the ER, especially in the smooth ER, than in extra-ER compartments. Thus, BaP is metabolized mainly through the phenol/quinone pathway in the ER and through the epoxide/diol pathway in other compartments, leading to accumulation of more phenolic metabolites and less diols/diones in microsomes than in the whole-cell. Data from the present report support this postulation. Specifically, we observed that overexpression of catalase upregulated BaP-induced expression of CYP1A1 and 1B1, but barely altered EH1 expression in microsomes. This undeniably diminishes the ratio of EH1 versus CYP1 proteins. Correspondingly, the ratio of BaP phenols versus diol/dione metabolites is increased in the microsomes obtained from catalase-transgenic cells than in those from WT cells.

The microscopy studies in this report show that the ER/whole-cell ratio of CYP1A1 was relatively lower under the control conditions. BaP treatment increased the ratio of CYP1A1 in the ER and the whole-cell. These findings suggest that BaP-increased CYP1A1 is retained in the ER compartment in MAECs. It has been suggested that CYP1 family enzymes are incorporated into the ER membrane in a signal recognition particle (SRP)-dependent manner during the translation process, with the catalytic site facing the cytoplasm. A small fraction of them, of which the catalytic site faces the lumen of the ER, escapes ER retention, and is transported via the Golgi apparatus to the plasma membrane [31]. The N-terminal residues of these proteins are responsible for anchoring them in the membrane. Modification of the N-terminal sequence, possibly by phosphorylation or proteolytic processing, prevents the ribosome-nascent chain complex binding the SRP. This fraction of CYP1 proteins are synthesized in the cytoplasm and imported into the mitochondria via the mitochondrial targeting signal [32, 36]. It has been reported that CYP1A1 proteins obtained from the microsomes were much more efficient than those obtained from the mitochondria with regard to BaP metabolism [27], and that BaP oral administration-induced toxicity in CYP1A1 knockout mice could be diminished by selective expression of CYP1A1 in the ER but not by selective expression of this gene in the mitochondria [36]. Thus, retention of CYP1A1 in the ER in response to BaP exposure could be important for BaP metabolism. It has been postulated that CYP1B1 may localize primarily in extra-ER compartments and is not tightly coupled to phase II XMEs. Activation of CYP1B1 therefore results in the BaP intermediate accumulation in cells [16]. In contradiction with this postulation, our data suggest that CYP1B1 localizes primarily in the ER of MAECs under the basal and BaP-treated conditions. Thus, CYP1A1 in MAECs might not be geographically closer than CYP1B1 to phase II enzymes. In addition, our data showed that overexpression of catalase augmented BaP-induced expression of CYP1A1 and 1B1, but did not affect its ER/whole-cell ratio. Thus, decrease in BaP intermediates under reduced oxidative conditions is not due to regulation of the ER retention of CYP1 proteins.

Benzo(a)pyrene detoxification reaction is catalyzed by GSTs, UGTs and SULTs, which add a glutathione, glucuronic acid or a sulfate to the functional group, converting BaP intermediates to hydrophilic conjugates. Glutathione S-Transferase Pi (GSTP) has been reported to be relatively more efficient than other classes of GSTs in conjugation of glutathione with BaP intermediates [37]. We previously reported that overexpression of catalase enhanced BaP-induced GSTP expression in MAECs. Though historically classified as a cytosolic protein, GSTP has been found in the mitochondria [38] and plasma membrane [39]. Here we observed that GSTP is detectable in the microsomes obtained from MAECs, and overexpression of catalase enhanced BaP-induced GSTP expression. Increase in expression of phase II enzymes, *e*.*g*., GSTP, could be a mechanism, which underlies the paradoxical findings that overexpression of catalase upregulates the expression of phase I enzymes CYP1A1 and 1B1, but lowers the BaP intermediates in MAECs. We previously observed that knockdown of AhR diminished BaP-induced GSTP expression, and elevated the accumulation of BaP reactive intermediates in MAECs overexpressing catalase. These observations suggest that overexpression of catalase upregulates phase II enzymes, such as GSTP, accelerating BaP detoxification via a mechanism involving AhR. Further studies are required to study whether overexpression of catalase induces other GST isoenzymes, as well as other phase II enzymes, such as UGTs and SULTs.

One of the mechanisms underlying the toxicity induced by the BaP phenols/quinone pathway is the redox cycling of quinones, semiquinones and quinols, which generates ROS, such as superoxide and H_2_O_2_ [6–8]. The phase II protein NQO1 bypasses the formation of semiquinones and prevents quinone-semiquinone-quinol redox cycles, thus reducing ROS generation. Data from studies using human cancer cells demonstrated that NQO1 distributes in the cytosol and nucleus, but not in the ER, mitochondria and Golgi [40]. However, a large amount of NQO1 protein has been found in the ER and mitochondria in several mouse tissues, such as the liver, kidney, lung and small intestine [32]. Here we observed that NQO1 was present in the microsomes obtained from MAECs, and overexpression of catalase increased BaP-induced expression of NQO1. Thus, catalase diminishes BaP-induced H_2_O_2_ not only by directly destroying H_2_O_2_, but also by upregulating NQO1 expression, which reduces H_2_O_2_ generation during BaP metabolism. We previously reported that the overexpression of catalase upregulates NQO1 expression by activation of transcription factors AhR and nuclear factor erythroid 2-related factor-2 (Nrf2) [18].

In summary, this report demonstrated that the spectrum of BaP intermediates in microsomes were different from that we previously observed in the whole-cell in MAECs [17], *i*.*e*., microsomes accumulate more BaP phenols than diols/diones after BaP treatment. Overexpression of catalase reduced all these BaP metabolites, with a greater magnitude of reduction in BaP diols/diones than in phenols. In addition, we observed that CYP1A1, 1B1 and EH1 were located mainly in microsomes, and NQO1 and GSTP were partially distributed in microsomes. BaP upregulated the expression of CYP1A1, 1B1 and NQO1 but not EH1 and GSTP in WT MAECs. Overexpression of catalase increased BaP-induced expression of these proteins in the following order: CYP1A1>NQO1>GSTP>CYP1B1>EH1. Preferential upregulation of CYP1A1 and phase II enzymes might be a mechanism for catalase to reduce BaP intermediates.
